# Therapeutic Effects of Systemic Administration of the Novel RANKL-Modified Peptide, MHP1, for Ischemic Stroke in Mice

**DOI:** 10.1155/2018/4637084

**Published:** 2018-07-30

**Authors:** Munehisa Shimamura, Hironori Nakagami, Hideo Shimizu, Kouji Wakayama, Tomohiro Kawano, Yuka Ikeda, Hiroki Hayashi, Shota Yoshida, Hideki Mochizuki, Ryuichi Morishita

**Affiliations:** ^1^Department of Health Development and Medicine, Osaka University Graduate School of Medicine, Japan; ^2^Department of Neurology, Osaka University Graduate School of Medicine, Japan; ^3^Department of Clinical Gene Therapy, Osaka University Graduate School of Medicine, Japan; ^4^Department of Advanced Clinical Science and Therapeutics, Graduate School of Medicine, The University of Tokyo, Japan; ^5^Department of Geriatric and General Medicine, Osaka University Graduate School of Medicine, Japan

## Abstract

Microglial healing peptide 1, “MHP1”, is a newly developed synthetic peptide composed of the DE and a part of the EF loop of the receptor activator of nuclear factor-кB (NF*κ*B) ligand (RANKL). Our previous report demonstrated that MHP1 significantly inhibits Toll-like receptor (TLR) 2- and 4-induced inflammation in microglia/macrophages through RANK signaling without osteoclast activation. However, its inhibitory effects on ischemic stroke when administered intravenously have not been clarified. First, we examined whether MHP1 could penetrate the brain parenchyma. Intravenous injection of FITC-conjugated MHP1 demonstrated that MHP1 could cross the blood-brain-barrier in peri-infarct regions, but not in intact regions. Because MHP1 in the parenchyma was reduced at 60 minutes after injection, we speculated that continuous injection was necessary to achieve the therapeutic effects. To check the possible deactivation of MHP1 by continuous injection, the anti-inflammatory effects were checked in MG6 cells after incubation in 37°C for 24 hours. Although the inhibitory effects for IL6 and TNF*α* were reduced compared to nonincubated MHP1, its anti-inflammatory efficacy remained, indicating that continuous administration with pump was possible. The single and successive continuous administration of MHP1 starting from 4 or 6 hours after cerebral ischemia successfully reduced infarct volume and prevented the exacerbation of neurological deficits with reduced activation of microglia/macrophages and inflammatory cytokines. Different from recombinant RANKL, MHP1 did not activate osteoclasts in the paralytic arm. Although further modification of MHP1 is necessary for stabilization, the MHP1 could be a novel agent for the treatment ischemic stroke.

## 1. Introduction

Our previous report demonstrated that the receptor activator of nuclear factor-кB (NF*κ*B) ligand (RANKL)/receptor activator of NF*κ*B (RANK) is a novel signal that regulates inflammation in microglia and macrophages (M/M) through Toll-like receptors (TLRs) 4 [1], which are important receptors for damage-associated molecular patterns in ischemic stroke [2]. M/M express both RANKL and RANK in peri-infarct regions, and an enhancement of the RANKL/RANK signal using recombinant RANKL (rRANKL) in osteoprotegerin knockout mice resulted in the reduction of ischemic injury [1]. Because recent clinical trials that have targeted the classical inflammation pathways with, for example, minocycline [3] and uric acid [4], have failed to show efficacy, we speculated that the regulation of TLR4 signals by enhancing RANK signaling using rRANKL might be a novel strategy to treat ischemic stroke [1]. However, the possibility of osteoclast activation and osteoporosis after stroke is a potential problem with the use of rRANKL; RANK is expressed in osteoclast precursors and the stimulation of RANK signaling by the systemic injection of rRANKL could induce osteoporosis [5]. To solve this problem, we have developed a novel peptide, “MHP1,” as a partial RANKL agonist that can reduce TLR2- and TLR4-induced inflammation without activating osteoclasts [6]. MHP1 does not include the binding sites of RANKL for its receptor, RANK, which is responsible for osteoclast activation (AA^″^ and/or CD loops [7, 8]), but includes binding sites unrelated to osteoclastogenesis (DE and EF loops). Interestingly, this peptide inhibited RANKL-induced osteoclast activation. The sequence of MHP1 is “LMVYVVKTSIKIPSSHNLMKGGSTKNWSGN”, but “LMVYVVKTSIKIPSS” is a key region for anti-TLR-induced inflammation [6]. A single intracerebroventricular injection of MHP1 was shown to significantly decrease ischemic stroke size when injected at 4 hours after transient middle cerebral artery occlusion (tMCAo) [6]. However, considering clinical utility, the systemic administration of MHP1 would be more feasible to treat ischemic stroke. Thus, we examined the ability of MHP1 to cross the blood-brain barrier (BBB) and the stability of MHP1 at 37°C for continuous injection of MHP1. We also examined its anti-TLR7- and TLR8-induced inflammation because a recent study showed that the activation of TLR7 and TLR8 are associated with poor outcome and greater inflammatory response in acute ischemic stroke [9]. Finally, the therapeutic effects and influences on osteoclast activation in the paralytic arm were examined after the systemic injection of MHP1 in tMCAo model.

## 2. Materials and Methods

### 2.1. Peptide Design and Synthesis

Synthetic MHP1 (NH2-LMVYVVKTSIKIPSSHNLMKGGSTKNWSGN-COOH) or FITC-conjugated MHP1 (FITC-C6-LMVYVVKTSIKIPSSHNLMKGGSTKNWSGN-COOH) was purchased from ILS, Inc (Ibaragi, Tsukuba, Japan), dissolved in ddH2O to make a 1 or 2 mg/mL solution, and stocked at 4°C until use.

### 2.2. Cell Culture and Enzyme-Linked Immunosorbent Assay (ELISA)

MG6 cells were obtained from RIKEN BRC (Tsukuba, Japan) [10, 11]. The MG6 cells were maintained in Dulbecco's Modified Eagle Medium (DMEM, Nakarai, Kyoto, Japan) supplemented with 10% fetal bovine serum (FBS, Thermo Fisher Scientific, Waltham, MA, USA), 10 *μ*g/mL insulin (Sigma-Aldrich, St. Louis, MO, USA), and 100 *μ*M 2-mercaptoethanol (Sigma-Aldrich). These cells (1 × 10^5^ cells) were plated in 96-well plastic culture dishes. After overnight culture, the medium was replaced with DMEM supplemented with 4% FBS. Lipopolysaccharide (LPS,* Escherichia coli* 0111:B4; Sigma-Aldrich, St. Louis, MO, USA) and MHP1 were added to the medium, which was then harvested at 24 hours after stimulation. For examining the stability at 37°C, MHP1 (1 mg/mL in ddH_2_O) was incubated at 37°C for 24 hours before being added to the medium.

The concentrations of TNF-*α*, IL-6, and IL-12/IL-23p40 were measured using commercially available ELISA kits: TNF-*α*, Quantikine Mouse TNF-*α* ELISA Kit (R&D systems); IL-6, Quantikine Mouse IL-6 ELISA Kit (R&D systems); Mouse IL-12/IL-23p40 ELISA kit (R&D systems).

### 2.3. Surgical Procedure

The animal studies were approved by the Animal Committee of Graduate School of Medicine, Osaka University (25-029-012), and all animal experiments were carried out in accordance with the guidelines of Osaka University. All surgeries were performed under isoflurane, and all efforts were made to minimize suffering. The C57/Bl6/J mice were obtained from CLEA Japan, Inc. The transient MCAo procedure was described previously [6]. Briefly, the mice were anaesthetized with isoflurane (1.4%). The cerebral blood flow (CBF) was measured using a laser Doppler flowmeter (Unique Acquisition software; Unique Medical, Osaka, Japan). A 6.0 monofilament surgical suture was advanced into the internal carotid artery to obstruct the origin of the middle cerebral artery. The filament was left in place for 40 minutes and then withdrawn. For all the mice, rectal temperature was maintained at 37.0 ± 0.5°C during the surgery and recovery period until the animals regained consciousness. Only animals that exhibited a typical reduction pattern and >82% reduction in CBF during MCAo, in which CBF recovered by 30–80% after 5 minutes of reperfusion, and modified Bederson scale [12] at 4 or 6 hours after ischemia were included in the study. MHP1 (4 mg/mL in water) was diluted to 2 mg/mL in saline, and 150 *μ*L of MHP1 was injected intravenously at 4 or 6 hours after MCAo induction. MHP1 (200 *μ*l, 2 mg/mL) was subsequently injected subcutaneously using an Alzet mini-osmotic pump (2001D, DURECT Corporation, Cupertino, USA) for 24 hours. As a control for MHP1, 0.45% saline was injected in a similar manner.

The ischemic damage was evaluated at 48 hours after MCAo induction in sections stained with cresyl violet. Coronal sections (12 *μ*m thickness) were made at –1.4, –0.7, 0, 0.7, and 1.4 mm from the bregma, mounted on the stereomicroscope, and photographed. The corrected hemispheric lesion area (HLA) was calculated as follows: HLA (%) = [LT−(RT−RI)]/LT × 100, where LT is the area of the left hemisphere, RT is the area of the right hemisphere, and RI is the infarcted area. The left radial bone was also examined at 48 hours after MCAo induction and stained for Tartrate-resistant acid phosphatase (TRAP) to evaluate osteoclast activation. For TRAP-stained surface quantification, we measured the total length of TRAP^+^ surface (TL) and the surface length in the trabecular bone (SL) under the growth plate and calculated as follows: TRAP^+^ surface length (%) = [TL/SL] × 100. Percentage of TRAP^+^ surface length (%) above the averaged TRAP^+^ surface length (%) in the normal mice was calculated as TRAP^+^ surface length (%control).

### 2.4. Immunohistochemical Staining

The mice were perfused with 4% paraformaldehyde, and the brains were cut into 12-*μ*m thick sections. These sections were fixed and then blocked. The sections were incubated with anti-F4/80 (1:50; AbD Serotec, Oxford, UK) or anti-FITC antibody (1:200; Abcam, Cambridge, UK). Then, the sections were incubated with an anti-rat fluorescent antibody (1:500, Alexa Fluor 488; Invitrogen) or biotinylated anti-goat antibody (1:100, Vector Laboratories, Burlingame, CA, USA). Tyramide signal amplification (TSA) plus system (PerkinElmer, Waltham, MA, USA) was used in the immunohistochemistry for FITC. The immunohistochemical staining was examined using a fluorescence microscope (FSX-100; Olympus, Tokyo, Japan) or confocal microscopy (FV10i FLUOVIEW; Olympus).

### 2.5. Real-Time Reverse Transcription Polymerase Chain Reaction (RT-PCR)

The ischemic hemisphere was collected at 48 hours after MCAo. The mRNAs were isolated using QIAGEN RNeasy Lipid Tissue Mini Kit (Qiagen, Germantown, MD, USA), according to the manufacturer's recommendations. The cDNA reaction was performed using a High-Capacity cDNA Archive kit (Applied Biosystems) according to the manufacturer's instructions. The oligonucleotide primers used exclusively in the in vitro experiments were purchased according to the following identification:* MCP1*: Mm00441243;* IL-6*: Mm00446190;* Arg1*: Mm00475988;* iNOS*: Mm00440502;* GAPDH*: Mm99999915 (Applied Biosystems). The 5' nuclease assay PCRs were performed in a MicroAmp Optical 384-well reaction plate using an ABI PRISM 7900 Sequence Detection System. The levels of the target genes were quantified by comparing the fluorescence generated by each sample with that of the serially diluted standard, and the target gene expressions were normalized by the level of GAPDH expression in each individual sample.

### 2.6. Statistical Analysis

All values are expressed as the mean ± standard deviation (SD). Multiple comparisons were evaluated by analysis of variance (ANOVA) followed by Dunnett's multiple comparison test. Two-way ANOVA followed by Dunnett's multiple comparison test was performed in neurological severity score. Differences were considered to be significant at* P*<0.05. All statics were calculated using GraphPad Prism software version 6.07 (GraphPad, Inc., San Diego, CA, USA).

## 3. Results and Discussion

**Figure 1:**
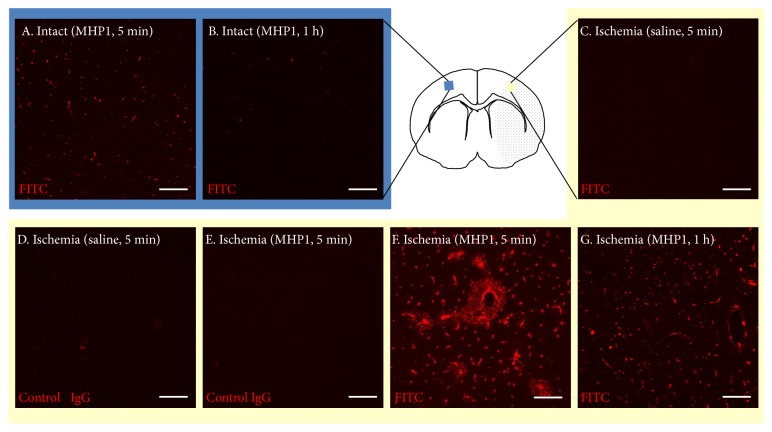
**Penetration of FITC-conjugated MHP1 in the ischemic brain**. FITC-conjugated MHP1 was injected intravenously 4 hours after ischemia and observed 5 min (A, E, F) or 1 hour (B, G) later. Saline was injected as a control (C, D). At 5 min after injection of FITC-conjugated MHP1, FITC was expressed only in the microvessels of intact regions (A). In ischemic regions, the expression was observed in the parenchyma, especially around microvessels (F, arrows). At 1 hour after ischemia, FITC was predominately absent from the intact regions (B) but was still observed in the ischemic regions (G); however, the signal was less than that of the corresponding 5-min section (F, G). No signal was observed in the sections stained with an anti-FITC antibody for saline-treated mice (C), the sections stained with control IgG for saline-treated mice (D), and MHP1-treated mice (E). Bar = 100 *μ*m.

**Figure 2:**
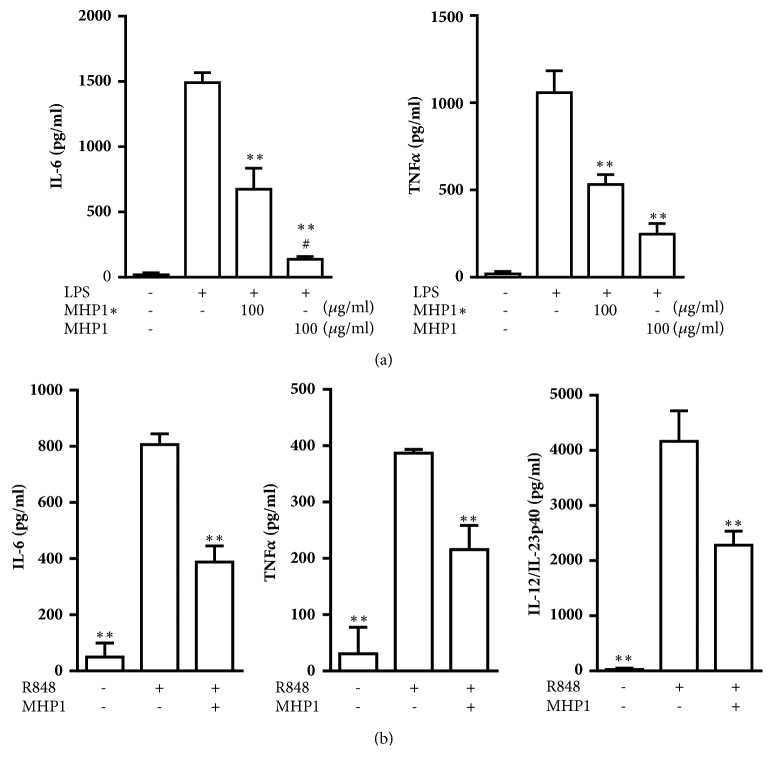
**Stability and inhibitory effects for TLR7- and TLR8-induced inflammation in MG6 cells**. (a) MHP1 solution was incubated at 37°C for 24 hours and added to the medium (MHP1*∗*) containing lipopolysaccharides (LPS) in MG6 cells. Expression of IL-6 and TNF*α* in the medium was analyzed. *∗∗P*<0.01 versus LPS-treated cells; #*P*<0.05 versus LPS and incubated MHP1-treated cells. (b) The anti-inflammatory effects of MHP1 were analyzed in cells, which were stimulated with TLR7 and TLR8 agonist, R848. *∗∗P*<0.01 versus R848-treated cells.

First, we examined whether MHP1 could penetrate the brain using FITC-conjugated MHP1. Immunohistochemistry for FITC showed that FITC was observed in microvessels of intact brain regions at 5 minutes after injection of MHP1 (Figure 1A), whereas it was not as prevalent 1 hour after injection (Figure 1B). Immunoreactivity was not seen in saline-treated mice (Figure 1C). However, in the ischemic regions, FITC was observed in the cerebral parenchyma, especially around the vessels of the ischemic region 5 minutes after injection (Figure 1F). As negative controls for immunohistochemistry for FITC, saline- or MHP1-treated mice was stained with control IgG, but no fluorescence was observed (Figures 1D and 1E). FITC in the injured parenchyma faded at 60 minutes after injection but was still observed in the vessels (Figure 1G). These results suggested that systemically injected MHP1 could be delivered to the cerebral parenchyma across the BBB in the ischemic regions, but MHP1 in the parenchyma was reduced at 60 minutes after injection. From these results, we speculated that MHP1 should be administered continuously after the injection of one bolus to keep it within the target lesion. To administer MHP1 continuously, we investigated whether MHP1 was still effective after incubation at 37°C for 24 hours because the maximum duration of Alzet pump with maximum dose is 24 hours. MHP1, which was incubated at 37°C for 24 hours, was added to the MG6 cells, followed by the treatment with LPS for 24 hours. Although the inhibitory effects for IL6 and TNF*α* were reduced compared to the MHP1, which was not incubated, its anti-inflammatory efficacy was remained (Figures 2(a) and 2(b)). This observation indicated that MHP1 released from Alzet pump at 24 hours after implantation was still effective in inhibiting TLR4-induced inflammation.

We next examined the effects of MHP1 for TLR7- and TLR8-induced inflammation. Stimulation with TLR7 and TLR8 agonist, R848, increased the expression of IL-6, TNF*α*, and IL12/IL-23p40 in MG6 cells, but MHP1 suppressed the production of these inflammatory cytokines (Figure 2(b)).

**Figure 3:**
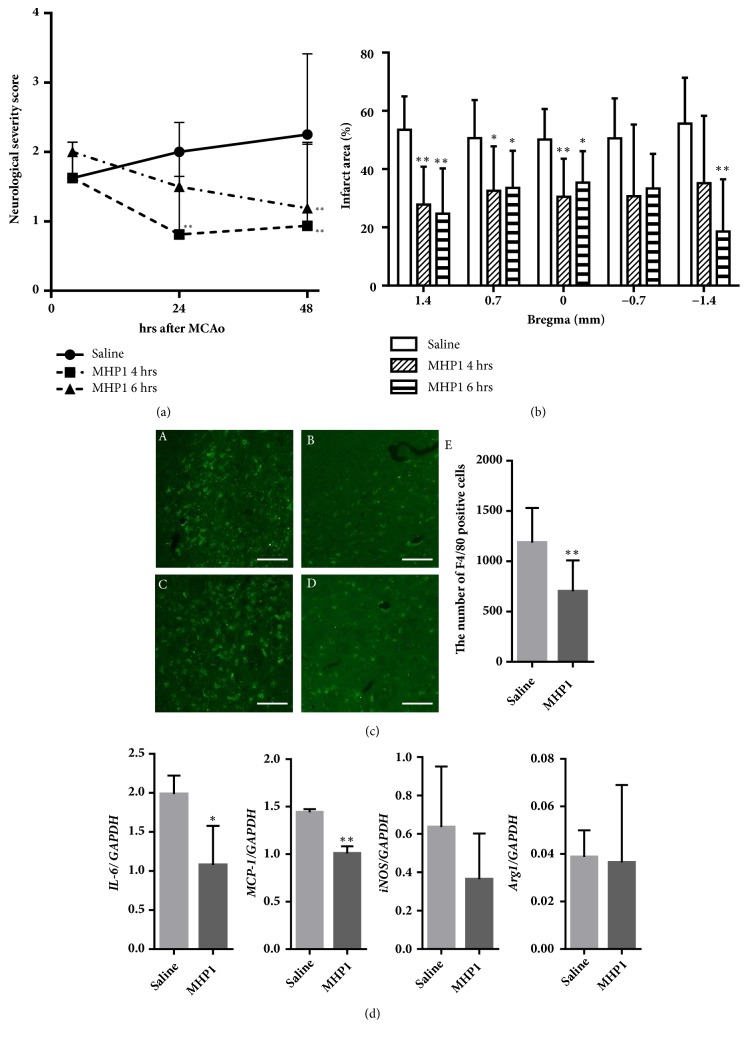
**Effects of MHP1 after transient middle cerebral artery occlusion**. (a) MHP1 was injected intravenously at 4 or 6 hours after middle cerebral artery occlusion with successive continuous subcutaneous injection for 21 hours. Neurological deficits improved 24 hours after administration of MHP1 at 4 hours after ischemia. Mice treated at 6 hours after ischemia showed a delayed improvement. (b) Infarction areas at 48 hours after ischemic insult. MHP1 *∗ P*<0.05 and *∗∗P*<0.01 versus the saline group. n = 8 in each group. (c) Typical images of F4/80 positive cells in the peri-infarct region in cerebral cortex (A, B) and caudate putamen (C, D) in saline-treated mice (A, C) and MHP1-treated mice (B, D). The number of F4/80 positive cells were quantified in the whole area of infarct region (n = 7 in each group). *∗∗P*<0.01 versus the vehicle group. (d) Expression of* MCP-1*,* IL-6*,* iNOS*, or* Arg1 *mRNA in infarct hemisphere (n = 3 in each group). *∗ P*<0.05 and *∗∗P*<0.01 versus the vehicle group.

Next, we examined whether the systemic administration of MHP1 could be protective in ischemic injury. As previously mentioned, intracerebroventricular injection of MHP1 was effective at 4 hours after the insult. Thus, we first investigated whether the systemic administration of MHP1 at 4 hours after ischemia was effective. When the maximum dose of MHP1 was intravenously injected and followed by continuous subcutaneous injections, MHP1 significantly prevented both the expansion of the infarct volume and the exacerbation of neurological deficits (Figures 3(a) and 3(b)). Because RANK increases at 4 hours to a maximal expression at 12 hours after ischemia [1], we speculated that we could extend the therapeutic time to at least 6 hours. As expected, systemic administration of MHP1 was also effective when the treatment was administered 6 hours after the insult (Figures 3(a) and 3(b)). To check whether MHP1 could inhibit inflammation and microglia activation, we examined the number of F4/80 positive cells in the ischemic region and the expression of* IL-6 *and* MCP-1* mRNA at 48 hours after the treatment. The number of F4/80 positive cells and the expression of* IL-6* and* MCP-1* was lower in the mice-treated with MHP1 (Figures 3(c) and 3(d)). We also checked whether MHP1 could influence on M/M phenotype after ischemic insult, but there were no significant differences in M1 marker mRNA,* iNOS*, and M2 marker,* Arg1* (Figure 3(d)). This is compatible with the previous paper showing that treatment with recombinant RANKL did not influence M/M phenotype at 24 hrs after ischemic injury [1]. Considering that ischemic injury induces early increase of M2 phenotype from 1 to 3 days, followed by a transition to M1 phenotype from 3 days [13], further studies are necessary to clarify whether MHP1 might affect M2 to M1 transition in the late stage of ischemic injury.

**Figure 4:**
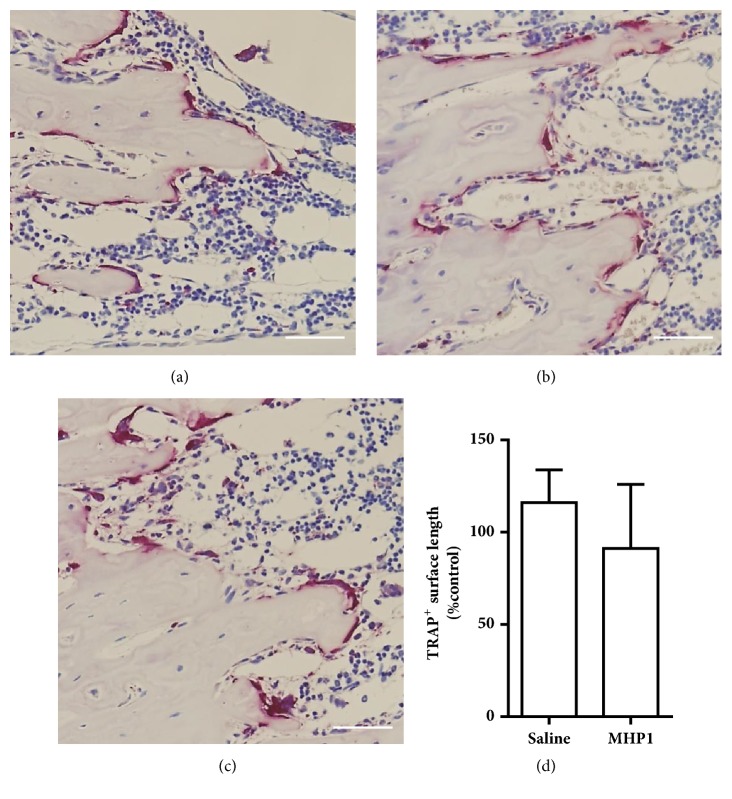
**Effects of MHP1 on osteoclast activation**. Tartrate-resistant acid phosphatase (TRAP) staining of the radial bone in the right forelimbs in normal mice (a) and paralytic forelimbs of saline-treated (b) or MHP1-treated (c) MCAo mice. Bar = 50 *μ*m. Quantitative analysis of the TRAP-positive surface in the radial bone (d). Treatment with MHP1 showed a tendency of reduction of TRAP-positive surface length although there were no significant differences between saline- and MHP1-treated mice. n = 4 in each group.

Finally, we examined whether MHP1 would affect osteoclast activation in the radial bone of paralyzed forelimbs because we previously reported that MHP1 could inhibit RANKL-induced osteoclast differentiation [6]. TRAP staining showed that osteoclast activity was increased in saline-treated MCAo mice (Figure 4(a)) compared to normal mice (Figure 4(b)). There was a tendency of inhibition of the osteoclasts activation in mice treated with MHP1 although no significant differences were seen (Figures 4(c) and 4(d)). These results indicated that MHP1 at least did not activate osteoclasts in the paralytic arm and might be able to suppress osteoclasts activation.

Thus, we demonstrated that systemically administered MHP1 penetrated ischemic brain regions and significantly decreased the ischemic injury until 6 hours after the insult. In general, substances with a molecular weight <400 Da that form <8 hydrogen bonds can pass the BBB via lipid-mediated free diffusion [14]. Because the molecular weight of MHP1 is 3277.8 Da, it could not pass the intact BBB. However, it could cross the BBB in ischemic regions due to a breakdown in the BBB that has been reported to begin 2 hours after cerebral ischemia in rodent models [15]. The ability to cross the disrupted BBB in the ischemic region, but not in the intact region, is ideal for the treatment of ischemic stroke as it prevents unwanted effects on the intact brain. In clinical, patients examined with dynamic contrast-enhanced MRI at 1.3–90.7 hours after ischemic stroke showed increased permeability of BBB [16], which may indicate that MHP1 might be able to penetrate ischemic brain parenchyma in human. Considering that MHP1 is smaller than albumin, which could pass BBB due to the increased number of endothelial caveolae and transcytosis rate without structural defects in the acute stage of ischemic stroke [17], MHP1 might be also transported to the parenchyma through transcytosis. The penetrated MHP1 acted on the activated M/M in the peri-infarct regions, resulting in a reduction of ischemic injury through the inhibition of TLR2-, TLR4-, TLR7-, and TLR8-induced inflammation via RANK signaling because the expression of RANK was reported to be increased in activated M/M in peri-infarct regions from 4 hours to 12 hours after ischemia [1].

We previously reported that MHP1 dissolved in ddH_2_0 was stable at 4°C and retained anti-TLR signaling activity after 6 months [6]. However, in the present study, 24 hours after incubation at 37°C, the activity of MHP1 was reduced. This is probably because MHP1 includes methionine and tryptophan, which can be major sites of oxidization [18]; oxidized MHP1 might lose its anti-TLR activity. Modification of these peptide to reduce oxidization, such as a substitution with D-amino acids, or an addition of antioxidant might be a way to preserve its activity by inhibiting oxidation.

Clinically, osteoporosis is a serious complication after stroke [19] and stroke is associated with a 2.0-fold increase the risk of hip/femur fracture, which increased 5.1-fold among patients younger than 71 years old [20]. Prospective studies examining biochemical markers of bone turnover in hemiplegic patients [21] or ischemic stroke model in rats [22] suggest an early (within 7 days) increase in bone resorption after stroke although there is no direct evidence of its association with RANKL. Because morbidity and mortality from hip fractures might be reduced by preventing bone loss at an early stage [23], a tendency of decrease of TRAP-stained cells in paralytic forelimb in mice treated with MHP1 might be preferable in the treatment of ischemic stroke. Long-term treatment with MHP1 during the chronic stage of ischemic stroke might clarify the effects of MHP1 for inhibiting osteoporosis after stroke.

One limitation in the present study is that continuous subcutaneous administration of MHP1 with Alzet pump is impossible in clinical use. Because MHP1 will be administered intravenously followed by daily single subcutaneous injection or continuous intravenous injection clinically, further studies are necessary to clarify whether such methods of administration are effective.

## 4. Conclusions

In summary, this study showed that systemic administration of MHP1 penetrated cerebral parenchyma of ischemic regions and prevented ischemic injury and osteoclast activation in the paralytic arm. Treatment with MHP1, by targeting RANKL/RANK signaling, might become a promising approach to ischemic stroke, although further studies are needed to elucidate a more stable peptide.

## Data Availability

The data used to support the findings of this study are available from the corresponding author upon request.
